# Texas State Medical Association—Committee on Collective Investigation of Disease—Circular from the Chairman

**Published:** 1886-09

**Authors:** Sam. R. Burroughs

**Affiliations:** Chairman; Raymond, Texas


### SOCIETY NOTES.

### TEXAS STATE MEDICAL ASSOCIATION.

OFFICE COLLECTIVE INVESTIGATION COMMITTEE.

RAYMOND, Texas, July, 1886.

### SUPPLEMENTARY NOTES ON DIPHTHERIA.

The object of the inquiry is to secure information as regards the following points:

*1. The specific and general conditions which develop diphtheria, and the means by which it is communicated.*

*2. The relation sustained by diphtheria to other diseases.*

*3. The clinical history of the malady as determined by the individual attacked.*

It is deemed quite unnecessary to revert to the fact that our knowledge of the etiology of the various infectious diseases, when subjected to a process of critical elimination, is quite limited ; it is therefore desired that every fact standing in relation to the cause of diphtheria be carefully and accurately recorded after a thorough test as to its claim.

It is a well known fact that diphtheria displays many points in common with other diseases. With scarlatina, in its incubative period ; in the fact that it marks gradations from the mildest to the most malignant type ; in point of incidence—the seat of inflammation; in the gravity of the blood-poisoning and prostration in its severest form; and in the nephritis which not infrequently attends as a complication or sequel. It also resembles typhoid fever in the fact of its communicability from person to person, as well as its development from foul exhalations. It likewise bears similarity to small-pox and scarlet fever, since it may be propagated not only by contact or inoculation, but through the medium of the atmosphere.

Much attention should also be directed to the incubation period of this disease, and it should be borne in mind that "those cases are of the greatest value in which the receiver of infection is exposed to it but for a limited time, and all other sources of infection can be eliminated." The attention of the investigator is called to the possibility of a simple sore-throat being cultivated up to well-marked diphtheria, by being transmitted through several persons residing in localities and under circumstances favorable to its development. It is believed that diphtheria occurs more frequently in rural districts than in densely populated towns and cities. Information that may clear up doubts and establish truth in regard to this point, will be valuable.

Does a first attack impart any protection against a second ; or, does a family predisposition to throat affections determine a special liability to an attack from diphtheria ? Facts bearing directly on these points are especially desired.

The committee are especially anxious that the cards already in the hands of the members be filled and returned as early as may be practicable, otherwise no report can be made on the diseases represented—acute pneumonia, acute rheumatism and cholera. Unless special interest is developed and maintained in this work, by the members of the Association, your committee will be powerless.

Respectfully, your obedient servant,

SAM. R. BURROUGHS, Chairman.

[Readers will please observe the change in Dr. Burrough's P. O. address from Guy's Store to Raymond (same county, Leon)—Ed.]

